# Spurious and functional correlates of the isotopic composition of a generalist across a tropical rainforest landscape

**DOI:** 10.1186/1472-6785-9-23

**Published:** 2009-11-24

**Authors:** Terrence P McGlynn, Hee K Choi, Stefanie T Mattingly, Angela Upshaw, Evan K Poirson, Justin Betzelberger

**Affiliations:** 1 Department of Biology, California State University Dominguez Hills,1000 E Victoria St Carson, CA 90747, USA; 2 Department of Biology, Occidental College, Los Angeles, CA, USA; 3 East Valley High School, Los Angeles Unified School District, North Hollywood, CA, USA

## Abstract

**Background:**

The isotopic composition of generalist consumers may be expected to vary in space as a consequence of spatial heterogeneity in isotope ratios, the abundance of resources, and competition. We aim to account for the spatial variation in the carbon and nitrogen isotopic composition of a generalized predatory species across a 500 ha. tropical rain forest landscape. We test competing models to account for relative influence of resources and competitors to the carbon and nitrogen isotopic enrichment of gypsy ants (*Aphaenogaster araneoides*), taking into account site-specific differences in baseline isotope ratios.

**Results:**

We found that 75% of the variance in the fraction of ^15^N in the tissue of *A. araneoides *was accounted by one environmental parameter, the concentration of soil phosphorus. After taking into account landscape-scale variation in baseline resources, the most parsimonious model indicated that colony growth and leaf litter biomass accounted for nearly all of the variance in the δ^15^N discrimination factor, whereas the δ^13^C discrimination factor was most parsimoniously associated with colony size and the rate of leaf litter decomposition. There was no indication that competitor density or diversity accounted for spatial differences in the isotopic composition of gypsy ants.

**Conclusion:**

Across a 500 ha. landscape, soil phosphorus accounted for spatial variation in baseline nitrogen isotope ratios. The δ^15^N discrimination factor of a higher order consumer in this food web was structured by bottom-up influences - the quantity and decomposition rate of leaf litter. Stable isotope studies on the trophic biology of consumers may benefit from explicit spatial design to account for edaphic properties that alter the baseline at fine spatial grains.

## Background

It is generally thought that stable isotope ratios vary across terrestrial landscapes. The extent of this spatial variation is enigmatic, and not explicitly built into the design of experiments wherein stable isotopes of carbon and nitrogen are employed to infer the trophic biology of consumers [1]. Typically, a landscape may be sampled broadly, with samples pooled together based on the assumption that baseline isotopic ratios will not vary appreciably within one kilometer (e.g., [2,3]). Nevertheless, to our knowledge there has been no study to test whether baseline stable isotope ratios are consistent across short distances. Moreover, no study has heretofore elucidated the contribution of spatial variation in isotope ratios to the isotopic variation in the tissue of consumers, relative to the effects of biotic factors shaping spatial patterns in isotopic fractionation.

In a tropical rain forest, nearly half of the aboveground primary production falls to the ground as litter, soon to release its carbon and nutrients into the diverse detrital food web [4,5]. In tropical rain forest leaf litter, a warehouse of biological diversity, little is known about the functional biology of even the most abundant organisms [6]. The processes that govern detrital food web structure, particularly in tropical forests, are poorly known. Caution is required in the use of isotopes to draw inferences about the trophic position of animals across space, as there is great variance introduced by spatial heterogeneity in the baseline isotope ratios and unknown differences in rates of isotopic discrimination [7].

The leaf litter food web of tropical rainforests is replete with ants, the most ecologically dominant guild of predators in this environment. Most ants in the leaf litter are believed to be generalists in diet, consuming prey from a variety of trophic levels [8], and little is known about the environmental factors that may shape diet and isotopic discrimination. Investigations into the variation in the trophic ecology of generalists have focused on effects of heterogeneity in competition [9] as well as variation in the resource base itself [10].

Tropical forests demonstrate great heterogeneity in the density of arthropods within leaf litter. This heterogeneity tracks limiting nutrients, especially phosphorus [11,12]. Because animals are known to shift their diet in response to changes in resource availability, we may expect that spatial variation in the resource base may account for variation in prey selection by ants in leaf litter. Consequently, parameters of the resource base - including phosphorus, the biomass of leaf litter and the rate of its decomposition - may affect isotopic composition of higher-level consumers.

On the other hand, competitor density and diversity are more often considered to influence the trophic position of generalist animals (e.g., [13,14]). Animals with broad diets may shift their feeding to underexploited resources when faced with competition [15]. The effect of competitors on diet should covary with density and diversity along environmental gradients [16,17]. Tropical rain forest leaf litter demonstrates great heterogeneity in the density and diversity of ants, which compete with one another for energy and nutrients derived from the resource base, the litter itself [18,19]. Leaf litter ant densities correspond to characteristics of the resource base as well as microclimate and microhabitat [20,21]. Thus we might expect the density or diversity of competing ants to account for any variation in the isotopic composition of a litter-foraging ant species.

Stable isotopes are often used to infer the relative trophic position of animals and how trophic position varies across a landscape [1,22]. To establish relative trophic position of a single species across the landscape of a single forest, stable isotope approaches integrate the trophic position of the entire consumer chain, provided that the baseline isotopic signature is measured for each locality [2]. Shifts in the concentration of stable isotope of nitrogen, ^15^N, have been used to infer that generalist species have variable diets that respond to proximate environmental conditions [3], though it is unclear how environmental conditions altered the isotope ratio of baseline resources. Interspecific studies using ^15^N have confirmed that species of ants differ in trophic level, with some ant species occupying trophic positions similar to herbivores [23,24]. Tillberg et al. [25] tested for intraspecific δ^15^N variation in five species of ants. They found remarkably broad variation within a species, even within a single locality, suggesting that life history and small-scale environmental factors may interact to result in differences in the isotopic composition of ants.

In the present study, we evaluate the carbon and nitrogen isotopic composition of a generalist species, the gyspy ant *Aphaenogaster araneoides*, across the landscape of a single tropical rain forest. We demonstrate environmental parameters that robustly predict the isotope ratio of *A.araneoides*, and then account for landscape scale variation to test competing models to account for isotope discrimination factors.

## Results

**Figure 1:**
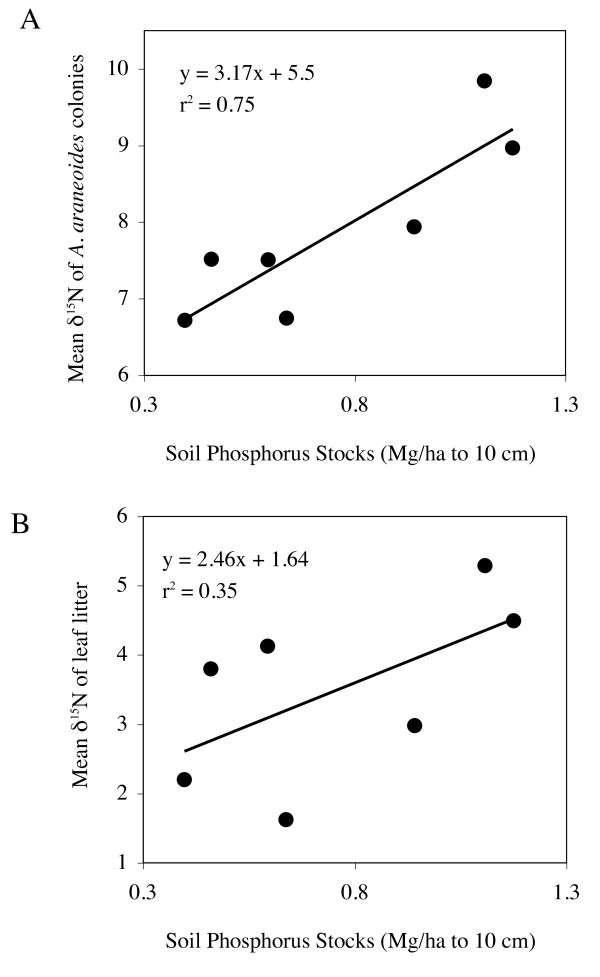
**Soil Phosphorus predicts δ^15^N of gypsy ants and δ^15^N of leaf litter**. Across a 500 ha. landscape, soil phosphorus stocks predicted mean δ^15^N of gypsy ants (panel A) and mean δ^15^N of the leaf litter, the resource base for gypsy ants (panel B).

Mean δ^15^N of *A. araneoides *from the seven sample sites within the La Selva forest ranged from 6.72‰ to 9.84‰, and mean δ^15^N of leaf litter ranged from 1.62‰ to 5.29‰, and the δ^15^N discrimination factor (relative to the leaf litter baseline) ranged from 3.71‰ to 5.12‰. Among the soil nutrients evaluated, P accounted for three quarters of the variance of δ^15^N in *A. araneoides *(Table 1; Figure 1a), consistent with the strong relationship between soil P and leaf litter δ^15^N across the landscape (Figure 1b). Mean δ^13^C in ant tissue ranged from -27.73‰ to -27.09‰, and from -31.57‰ to -28.02‰ in leaf litter. No soil nutrient accounted for more than a quarter of the variance in δ^13^C (Table 1). Among sampling sites, mean ant δ^13^C was independent of mean ant δ^15^N (y = 2.03 x + 63.2; F_1,5 _= 0.93; ns).

**Table 1:** Correlation (r) between soil parameters and isotopic composition of *A. araneoides*.

Soil nutrient	Correlation (r) with δ^15^N	Correlation (r) with δ^13^C
N	-0.18	-0.16

P	0.87	0.43

Ca	-0.14	-0.24

K	0.37	0.28

Fe	-0.10	-0.48

Mg	-0.38	0.05

Mn	0.22	0.39

Al	-0.20	0.02

Soil nutrients were evaluated as stocks in kg/ha to 10 cm depth. δ^15^N and δ^13^C values represent mean values of *A. araneoides *among seven sites.

Nearly all of the variance in the δ^15^N discrimination factor of *A. araneoides *relative to leaf litter was predicted by local colony growth (-) and mass of standing leaf litter (+) (the most parsimonious model; Table 2). The most parsimonious model for the C source of *A. araneoides *indicated that nearly all of the variance was predicted by colony size (+) and leaf litter decomposition rate (+) (Table 3).

**Table 2:** Regression models testing for the most parsimonious associations with the relative trophic position of *A. araneoides *(δ^15^N_*A. araneoides *_- δ^15 ^N_baseline_).

model (independent factors)	r^2^	SS	p	AIC
Leaf litter decomposition rate	0.056	0.13	0.610	-3.90

Log (mass, standing litter)	0.129	0.31	0.427	-4.47

Total soil P (0-10 cm depth)	0.129	0.31	0.430	-4.86

*Colony size*	0.277	0.66	0.225	-5.77

*Colony growth*	0.551	1.32	0.060	-9.11

Ant density	0.157	0.38	0.379	-4.70

Ant richness	0.206	0.49	0.307	-5.11

*Colony growth*, leaf litter decomposition rate	0.554	1.33	0.200	-7.15

*Colony growth*, log (mass, standing litter)	**0.978**	2.35	**0.0005**	-28.27*

*Colony growth*, total soil P	0.583	1.40	0.174	-7.62

*Colony growth*, litter-ant density	0.561	1.35	0.192	-7.27

*Colony growth*, litter-ant richness	0.571	1.37	0.184	-7.43

The most parsimonious model is indicated by *. Negative relationships with relative trophic position are indicated by italics.

**Table 3:** Regression models to test for the most parsimonious associations with (δ^13^C_*A. araneoides *_- δ^13^C_baseline_).

Model (independent factors)	r^2^	SS	p	AIC
Leaf litter decomposition rate	0.216	0.07	0.293	-19.66

Log (mass, standing litter)	< 0.01	< 0.001	0.987	-17.96

Total soil P (0-10 cm depth)	0.183	0.06	0.339	-19.36

Colony size	0.329	0.10	0.179	-20.74

Colony growth	0.002	< 0.001	0.917	-17.97

Litter-ant density	0.003	0.03	0.908	-17.98

Litter-ant richness	0.014	0.004	0.799	-18.06

Total soil P, colony size	0.394	0.12	0.366	-19.47

Total soil P, leaf litter decomposition rate	0.399	0.12	0.361	-19.52

Leaf litter decomposition rate, colony size	**0.907**	0.28	**0.009**	-32.61*

Total soil P, leaf litter decomposition rate, colony size	0.929	0.28	0.032	-32.42

The most parsimonious model is indicated by *. All significant relationships were positive.

## Discussion

The variation in δ^15^N and δ^13^C in *A. araneoides *within this 500-ha forest landscape was of equivalent magnitude to that reported from other ant species that were sampled over a much greater geographic extent by Tillberg et al [25]. It was surprising to find that soil phosphorus was such a tight predictor of the δ^15^N of *A. araneoides*, considering the proximity of sampling sites and multiple unknown trophic links between soil and this common predator. We found this relationship occurred because the variance in phosphorus throughout the 500 ha. forest was predictive of δ^15^N of leaf litter, which is the base of the food web for *A. araneoides*.

Across this forest landscape, the standing stocks and rates of decomposition of the leaf litter were principal determinants of the isotopic composition of gypsy ants. If one assumes that δ^15^N discrimination indicates relative trophic position and that the trophic shift of ^15^N is about 3, then gypsy ants range from one to two trophic steps above leaf litter, with a higher trophic position positively associated with the biomass of standing leaf litter. Such assumptions about δ^15^N discrimination may not withstand continued scrutiny [7]. Regardless, the rate of decomposition of leaf litter at each sample site was the environmental parameter that best accounted for the source of C in the diet of the local gypsy ants. These findings suggest that the basic properties of leaf litter, and the abiotic and biotic factors that govern these properties in tropical rain forests, are essentially regulating the isotopic composition of a generalist predator in the complex environment of the leaf litter food web.

The degree of δ^15^C discrimination from litter to ants was slight, though the variation that occurred was well accounted by environmental parameters. We found that ants with greater δ^13^C discrimination factors came from sites with faster leaf litter decomposition and greater mean colony sizes. We can infer that the source of C varies across the fertility gradient, though we will have less solid conclusions about the steps in the food web that can account for this variation, as the fractionation of C in litter food webs is open to speculation. Carbon experiences variable rates of fractionation depending on the nature of the trophic step [26]. As the rate of decomposition is expected to be closely tied to rates of microbial respiration, we may expect that the weighted source of C in the litter food web - from air, litter and soil - may vary along the decomposition gradient. Complicating matters further is the possibility that δ^13^C varies along a vertical gradient, because at the ground level much of the CO_2 _assimilated by leaves is of respiratory origin, but in the canopy much of the CO_2 _fixed in photosynthesis is of atmospheric origin. The C in forest-floor leaf litter is from a mélange of leaves from all vertical strata, and we are not able to create a defensible mixing model to evaluate C source. Nevertheless, because δ^15^N and δ^13^C tightly covaried among species of collembola along another decomposition gradient [10], we might surmise that the differences in δ^13^C among sites may be attributed to differences in trophic structure above the level of primary consumers. This might explain the association with colony size, as nutritional demands of the colony may change with age [8].

The present results reinforce the view that stable isotope ratios for a given species - regardless of its diet or other habits - are apt to vary across space. We have found that much of this variance may be attributed to ambient variation in the isotopic composition of the base of the food web. In this circumstance, the discrimination factor of δ^15^N is governed by P abundance. The mechanism behind the spatial coupling of P and δ^15^N remains open to speculation. Two non-competing possibilities include differential rates of N fixation and differential physiological rates of N fractionation under conditions of P limitation [27].

We found that the N stable isotope ratio within gypsy ant tissue varied across the range of 4 per mil, a magnitude on par with similar results for other ant species, from collections spanning much larger geographic areas [25]. After accounting for the baseline isotopic composition at each locality within the forest, we found that dietary breadth was not as broad as suggested by the ant-tissue δ^15^N values alone. This finding suggests that comparisons of δ^15^N for sessile organisms (or organisms that do not move large distances) must be constrained to very short distances unless baseline data are collected from each locality.

## Conclusion

Phosphorus limitation closely tracked the δ^15^N of gypsy ants because the δ^15^N of the base of the food web varied predictably with phosphorus at a fine spatial scale. The δ^15^N discrimination factor of gypsy ants was most parsimoniously explained by the mass of leaf litter on the forest floor. Spatial variation in δ^13^C in ants, relative to the resource base, was almost wholly accounted by colony life history and the rate of leaf litter decomposition. In terrestrial environments, the failure to incorporate fine-scale information on the isotopic composition of baseline resources may erroneously identify environmental factors predicting trophic position.

## Methods

The study was carried out along a well-documented soil-fertility gradient within the old-growth lowland tropical forest at La Selva Biological Station, in N.E. Costa Rica. This forest (50-250 m elevation) receives ca. 4 m of rain annually [28]. More information about La Selva is available at http://www.ots.ac.cr. We sited our studies at seven forest plots of the CARBONO Project plot network [29,30], chosen to represent the broadest range of ant density, ant nest sizes and soil nutrient concentrations in this landscape, based on previous studies [11,21]. The plots were 0.5 ha and were located across ca. 500 ha of the old-growth forest landscape.

At each of the seven sampling sites, three colonies of the gypsy ant, *Aphaenogaster araneoides*, were located by feeding a forager and following her back to her nest. Each colony was selected to be at least 10 m from all other colonies used in the study, to prevent overlap or adjacency of home ranges to ensure independence of sampling, based on known measures of home range area [31]. All colonies were collected in their entirety by excavation. We considered a nest to be fully excavated when we reached a discrete terminal chamber, typically containing early instar brood unattended by workers that had typically already been collected. Any nest that we judged to be not completely collected was excluded and another nest was collected in its stead. Freshly collected colonies were kept alive for at least eight hours inside re-sealable plastic bags before being frozen at -20°C for at least 24 h. We then counted all individuals in each colony to determine colony size (the number of adult workers) and an index of colony growth (the ratio of adult workers and worker pupae). After drying each colony at 60°C to constant mass, we took three replicate subsamples to characterize the colony with stable isotope analysis. Each subsample consisted of a thorax and six legs of a single individual ant selected to be ca. 1 mg to provide sufficient mass for accurate determination of isotope ratios. The gasters were intentionally excluded to prevent inclusion of residual food. For several other ant species, comparisons of stable isotope values between heads and thoraxes have indicated no significant differences between these tissue types [25].

The isotopic composition of the standing leaf litter at the time and site of each colony collection was used as the baseline of the food web for *A. araneoides*. At each ant sampling site, three forest-floor fine-litter samples (including leaf, twig, and reproductive materials), each ca. 10 L in volume, were collected from three haphazardly selected nearby locations. These samples were washed to remove soil and then dried at 60°C to constant mass. Each dried sample was ground to powder in a Wiley Mill and homogenized, three subsamples of ca. 2 mg were analyzed for isotopic composition, and the mean of these values was used to characterize each sampling site.

Stable isotope ratios are represented as δ^15^N and δ^13^C, representing per mil (‰) proportionality of heavy:light isotopes, relative to a universal standard for each. Samples were weighed into tin capsules in a microbalance to the nearest milligram. Ratios of stable isotopes in the ant tissue were measured with a PDZ Europa 20/-20 isotope ratio mass spectrometer at the UC Davis Stable Isotope Facility. For each sample, values of δ^15^N and δ^13^C were calibrated using values from established laboratory standards, run every 12 samples, calibrated against NIST Standard Reference Materials IAEA-N1, IAEA-N2, IAEA-N3, IAEA-CH7, and NBS-22.

We use (δ^15^N_*A*.*araneoides *_- δ^15^N_baseline_) as the δ^15^N discrimination factor of *A. araneoides*, in which the baseline is local (collection-site) standing leaf litter. The δ^13^C discrimination factor of *A. araneoides *was similarly evaluated, using (δ^13^C_*A*.*araneoides *_*- *δ^13^C_baseline_).

For each of the seven sampling sites, litter-dwelling ant density and richness were estimated with an "intensive sampling" protocol [32]. Ten 1 m^2 ^quadrats were established at 10 m intervals along a linear transect. Within each quadrat, all litter-ant nests were collected, sorted to species, and measured for size and growth estimates using the same method applied to *A. araneoides*. The species richness of the litter-ants was estimated using the mean number of species per quadrat; the density of litter ants was estimated using the mean number of adult litter ants nesting in quadrat.

We estimated the rate of leaf litter decomposition at each of the seven sampling sites with 2 successive 1-yr deployments of sets of litterbags containing a common litter, 10 g of freshly fallen and dried leaves of the pioneer tree *Cecropia obtusifolia*. Each litterbag was sized ca. 100 cm^2 ^and was constructed with 55 μm vinyl mesh. Three sets of five litterbags each were placed at each site in June 2006 and June 2007. Bags were sampled destructively in the following time sequence: 2 wk, 4 wk, 8 wk, 26 wk, 52 wk, for a total of six time points including t = 0. Using the mean values for each time step per plot, decomposition rate, k, was calculated based on the exponential rate of decay, e^-kt^, and for each site we averaged the resulting k values from the two measurement series (2006-2007 and 2007-2008).

Total nutrients in the surface soil were determined for each of the seven sampling sites (plots) based on compositing six regularly-spaced soil cores (0-10 cm depth) from each 0.5 ha plot. After being air-dried, sieved (2 mm) and ground, the samples were analyzed at the Institute of Soil Science and Forest Nutrition, University of Göttingen, Germany by HNO_3_-pressure extraction and ICP-AES (see [33]for details). While indices of available P have been found to be poorly related to ecosystem function at this site (e.g., [34], both stand structure and ecosystem processes significantly vary across the 3-fold within-forest range in total soil P at La Selva [30]. Total soil P was also previously shown to be a robust predictor of the variation in density of litter arthropods across the La Selva landscape [11].

To test the hypotheses that resource base, competition and life history are associated with trophic level and C source, we created multiple regression models for all variables with δ^15^N discrimination factor (δ^15^N_*A*.*araneoides *_*- *δ^15^N_baseline_) and δ^13^C discrimination factor (δ^13^C_*A*.*araneoides *_*- *δ^13^C_baseline_) as dependent variables. These multiple regression models should be interpreted cautiously given the number of comparisons and degrees of freedom. We used AIC [35] to select the factor(s) most closely associated with the dependent variable and to evaluate competing models for within-forest variance in the δ^15^N and δ^13^C of *A. araneoides*.

## Authors' contributions

TM wrote the manuscript, secured partial funding, and conducted fieldwork and labwork for all components. HC secured partial funding, and contributed to data analysis and interpretation. ST and JB contributed to research design and conducted fieldwork on litter sampling. AU contributed to research design and conducted labwork on ant samples. EP contributed to data analysis and conducted fieldwork on decomposition rates. All authors contributed to development of the manuscript and approve of the final version.
